# Recent advances in understanding cell types during human gastrulation

**DOI:** 10.1016/j.semcdb.2022.05.004

**Published:** 2022-05-21

**Authors:** Richard C.V. Tyser, Shankar Srinivas

**Affiliations:** 1 Department of Physiology, Anatomy and Genetics, South Parks Road, University of Oxford, Oxford, OX1 3QX, UK

## Abstract

Gastrulation is a fundamental process during embryonic development, conserved across all multicellular animals^1^. In the majority of metazoans, gastrulation is characterised by large scale morphogenetic remodeling, leading to the conversion of an early pluripotent embryonic cell layer into the three primary ‘germ layers’: an outer ectoderm, inner endoderm and intervening mesoderm layer. The morphogenesis of these three layers of cells is closely coordinated with cellular diversification, laying the foundation for the generation of the hundreds of distinct specialized cell types in the animal body. The process of gastrulation has for a long time attracted tremendous attention in a broad range of experimental systems ranging from sponges to mice. In humans the process of gastrulation starts approximately 14 days after fertilization and continues for slightly over a week. However our understanding of this important process, as it pertains to human, is limited. Donations of human fetal material at these early stages are exceptionally rare, making it nearly impossible to study human gastrulation directly. Therefore, our understanding of human gastrulation is predominantly derived from animal models such as the mouse^2,3^ and from studies of limited collections of fixed whole samples and histological sections of human gastrulae^4–7^, some of which date back to over a century ago. More recently we have been gaining valuable molecular insights into human gastrulation using *in vitro* models of hESCs^8–12^ and increasingly, *in vitro* cultured human and non-human primate embryos^13–16^. However, while methods have been developed to culture human embryos into this stage (and probably beyond), current ethical standards prohibit the culture of human embryos past 14 days again limiting our ability to experimentally probe human gastrulation. This review discusses recent molecular insights from the study of a rare CS7 human gastrula obtained as a live sample and raises several questions arising from this recent study that it will be interesting to address in the future using emerging models of human gastrulation.

## Historical background

Historically, insight into human gastrulation has relied on the morphological examination of fixed human embryos in collections such as the Carnegie^5^, Kyoto^6^, or Blechschmidt^7^ Collections. The Carnegie collection is one of the oldest and most well characterised. It has been used to establish the eponymous standardized staging system of human development. The collection was started in 1887 by Franklin Mall, who trained under Wilhelm His at the University of Leipzig in 1884. Wilhelm His was the first to write comparative descriptions of human embryos in the late 1800s. On Franklin Mall’s return to America, he began collecting human embryos and, in 1914, received $15,000 (the equivalent of approximately $400,000 today) from the Carnegie Institute for Science to begin characterizing in a scientific manner the normal and abnormal growth of embryos. This work was initiated at the newly formed Carnegie Institution of Washington Department of Embryology in Baltimore, where Mall had been made director^17^. Over the next fifty years the collection expanded to record more than 10000 embryos, which has served as the basis for hundreds of research articles and continues to be a valuable repository.

Whilst the embryos of this collection have proved invaluable for studies into human development, we must recognize that many of the early-stage samples were collected using practices that would be considered lax by current standards of ethics relating to human research. Embryos for the Carnegie collection were typically collected through hysterectomies on pregnant women who were not necessarily properly informed about the potential use of tissues and samples obtained during the surgery^18–21^. Pregnancy tests did not exist at the time, and while today it would be considered unethical to operate on a pregnant woman, this happened repeatedly until the 1950s.

The largest collection of human embryos is the Kyoto collection, initiated by Professor Hideo Nishimura at the Department of Anatomy, Kyoto University around 1961^6^. The two main factors leading to the initiation of this collection were the limited number of reliable samples being collected from spontaneous abortions among mothers with pathological conditions, and the revision of the Japanese Eugenic Protection Law in 1952, that allowed qualified gynecologists to terminate pregnancy for sociomedical reasons, leading to increases in the number of social termination of pregnancies^22^. This meant collection could be carried out in cooperation with obstetricians and 34,270 embryos and 3,852 fetuses where collected from 1962 to 1974^23^. The collection has since grown to over 44,000 human specimens. The Kyoto collection predominantly contains human embryos at later stages of development, characterizing both normal and abnormal development.

In terms of gastrulation unlike the Carnegie collection, the Kyoto collection only contains human embryos from CS7, that is, after the initiation of gastrulation, and has relatively few samples covering gastrulation stages (30 gastrulating embryos from 23,810 specimens as of 2014^24^). The rarity of human gastrulating embryos is also highlighted in the Carnegie Collection’s main research collection, called the “Yellow Files”, which contains 84 pre- and gastrulating staged embryos (CS2-9) compared to 555 post-gastrulation embryos. The relatively limited number of gastrulating embryos in both collections reflects the early stage at which human gastrulation occurs (between approximately 14 and 21 days post conception), as the majority of women are unlikely to know they are pregnant at this stage.

The samples in these collections are all fixed, and many are available only as sections. To make such valuable samples more readily available to the public online collections such as the Digital Embryology Consortium^25^ and The Virtual Human Embryo Project^26^ have been working to digitize the major embryology histological collections. Modern, non-destructive imaging technologies, such as magnetic resonance imaging (MRI), micro-computer tomography (micro-CT), and optical projection tomography (OPT), have been used to generate 3D models of human embryos at post-gastrulation stages based on these historical samples^23,27,28^.

More recently, the establishment of the Human Developmental Biology Resource in the UK has greatly facilitated fundamental research into all aspects of human development^29^. The HDBR serves as a vital resource which provides fresh as well as fixed embryonic and fetal tissue to researchers in line with the ethical guidelines laid out in the Polkinghorne Report (Review of the Guidance on the Research Use of Fetuses and Fetal Material, 1989). High-resolution episcopic microscopy (HREM) has been applied to human embryos collected through the HDBR, to generate high-resolution images of serial section of human embryos. These HREM data have been used to generate high resolution 3D models which capture great morphological detail. However, again due to lack of availability and size, this approach has only been applied to embryos from CS12/13 once gastrulation is completed. Therefore we are limited in our insight into the morphology of gastrulating human embryos to the historical sections of human gastrula reported in foundational studies such as those of Hertig and colleagues^4,5,30^.

## Staging the onset of gastrulation

**Figure 1:**
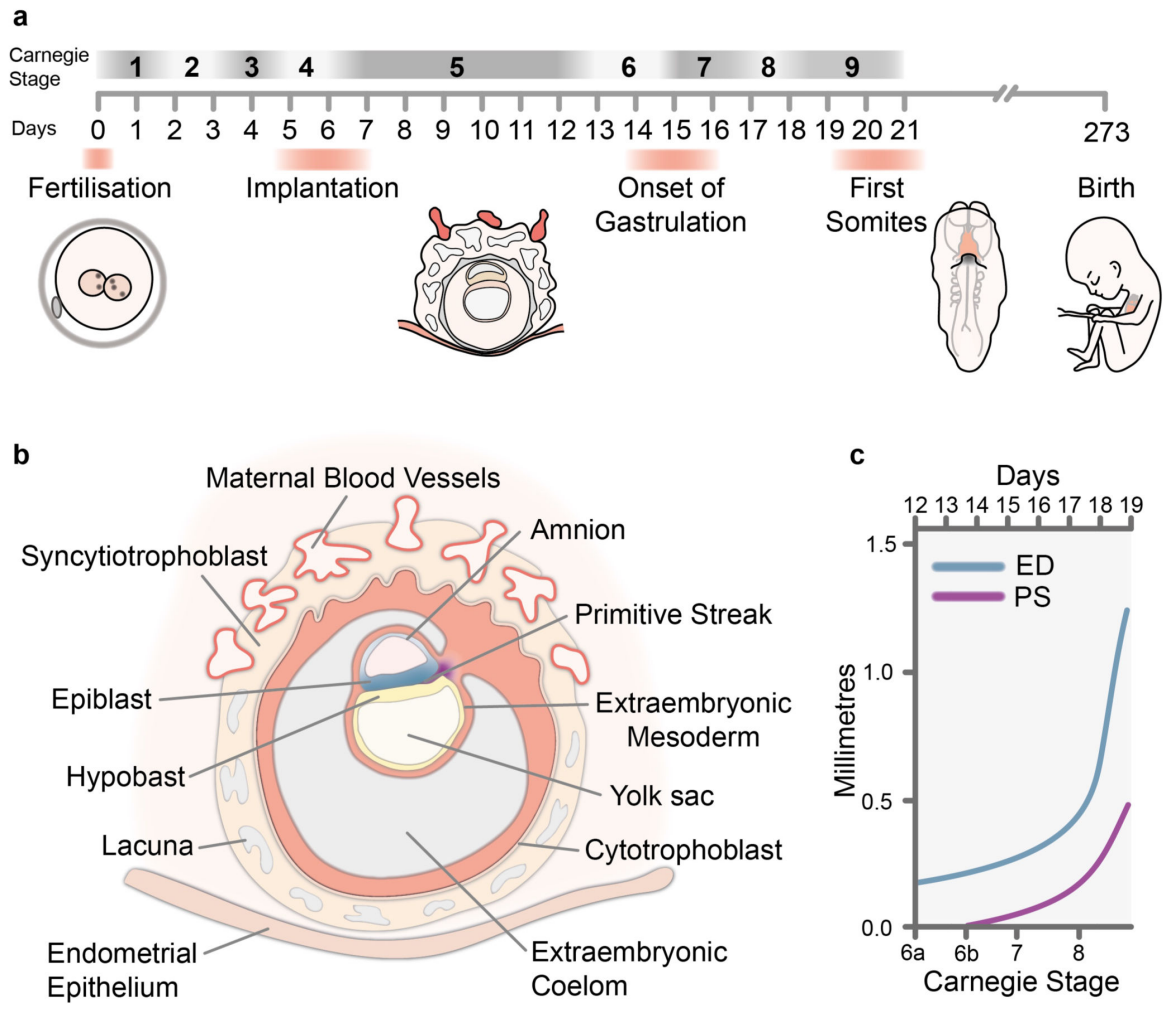
Staging Human Gastrulation a, Timescale of early human development highlighting Carnegie stages and key events during this period. b, Schematic diagram showing a human embryo at around Carnegie Stage 6b at the onset of gastrulation. c, Graph showing the onset of primitive streak formation and progression in size during Carnegie stages 6 to 8. Adapted from O’Rahilly 1973 Figure 23^5^. ED, Embryonic Disk; PS, Primitive Streak.

In order to accurately assess human development, and make comparisons with other model organisms, a standardized staging system is required. In 1942, George Streeter, using samples from the Carnegie collection, published his “Developmental Horizons in Human Embryos” which described criteria for early developmental staging^5^. Streeter’s Developmental Horizons represented 12 stages of human embryo development and became the basis for the Carnegie Staging system. Description of the Carnegie Stages (CS) 1 to 9, covering gastrulation, was first published in 1973^5^ (Figure 1a). Prior to 1973 there were two alternative staging systems put forward, both had pros and cons, but ultimately did not become established and the unified Carnegie staging is now the most widely used system.

**Figure 2:**
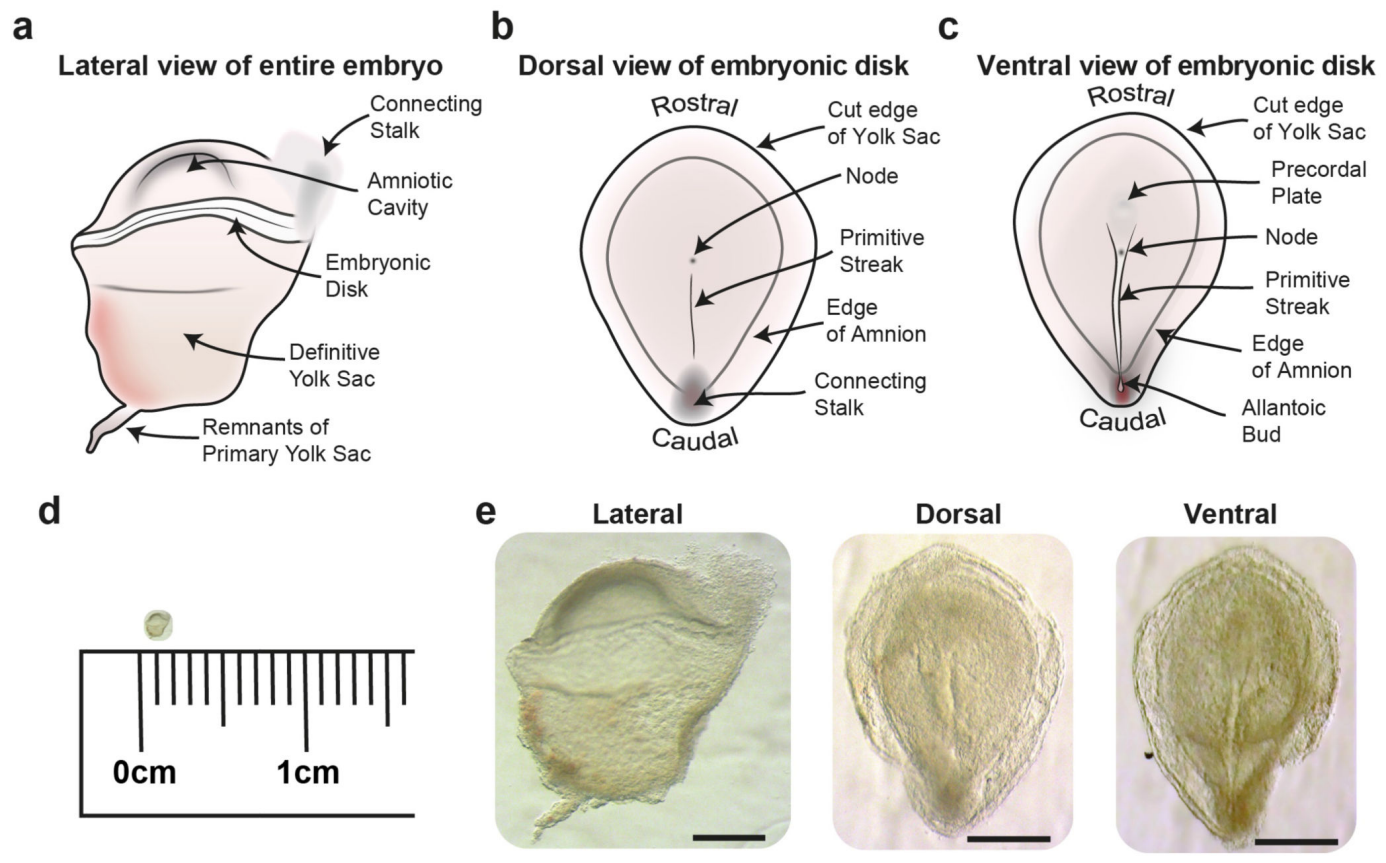
CS7 Human Gastrula a, Diagram showing a lateral view of an intact CS7 human embryo. Schematic diagrams of the dissected embryonic disk showing the primitive streak and node from a dorsal (b) and ventral view (c). d, Image of CS7 human embryo to scale with a 1cm ruler. e, Images of a CS7 human embryo. Left panel, lateral view of an intact embryo; middle panel, dorsal view of embryonic disk; right panel, ventral view of embryonic disk (Scale bar = 500μm).

During the time this staging system was established, in the absence of genetic markers, gastrulation was defined on the basis of morphology, by the formation of a primitive streak, the midline structure through which cells of the epiblast delaminate to form the endoderm and mesoderm. The primitive streak starts to form at the future caudal end of the embryonic disc and elongates to occupy roughly half the length of the disk, with a pit like feature, the node, at the rostral end of the streak (Figure 1b and 2). The node acts as an organizer of the primary body axis^31–33^, as well as sets up left-right axial asymmetries during development^34,35^(Figure 2). Once it has attained this peak length, the streak starts to regress back towards the caudal end of embryonic disk. While this is occurring, cells continuously delaminate through the streak to form endoderm and mesoderm.

Gastrulation in humans initiates during CS 6, a stage in part defined on the basis of the size of the embryo, with an embryonic disk typically between 0.15 to 0.45mm along the rostral-caudal axis and with the diameter of the chorion ranging from 1 to 4.5mm (Figure 1c). Embryos assessed at this stage were defined based on the presence of Chorionic Villi as well as axial features such as a primitive streak. Based on this, CS 6 was divided into two substages, CS 6a and CS 6b, with the latter corresponding with the visible presence of a primitive streak. At CS 6 all three extra-embryonic spaces; amniotic cavity, primitive yolk sac and Chorionic cavity are present. In CS 6b embryos the primitive streak is clearly defined and ranges in length from 0.021mm (Liverpool I embryo)^30^ to 0.187mm (HEB 18)^36^ from the caudal edge of the embryonic disk.

Historically the presence of a primitive streak was defined by Brewer’s Criteria^37,38^. These criteria included; active proliferation of cells, loss of the basement membrane separating the epiblast and endoderm, migration of epiblast cells and intermingling of the cells of the epiblast and endoderm disk. However the shape in which the human primitive streak manifests is not clear and therefore, there has been debate over whether some specimens contain a PS or not. This has led to some CS6a embryos being assessed as having a primitive streak and some CS6b embryos described as not having a PS. For example Brewer described the primitive streak as a crescent of cells at the caudal margin of the embryonic disc of a CS6b sample^37^. However this was contested in a follow up study of the same specimen^39^. In the chick, formation of the PS is induced by a region named Koller’s sickle, which is a crescent shaped thickening of cells at the caudal boundary of the epiblast^40^. The early streak then takes the form of a triangular structure in the same region before elongating towards the middle of the disc^1,41^. These early features would be difficult to detect based on morphology alone in human samples and even in model systems, are most readily detected at incipient stages based on the expression of genes such as Brachyury, which marks the streak in both mouse and chick^42,43^. One could speculate that what Brewer described as a crescent PS might be the equivalent in the human of the chick Koller’s sickle, which would put this CS6b specimen at slightly prior to streak formation. However, it is important to note that the rabbit, that also has a bilaminar disk embryo prior to gastrulation, does not show a structure similar to Koller’s sickle^44^.

In some young CS6b samples, the PS was describe as taking the form of a node, located in the middle of the embryonic disc. This led to debate as to whether the axial features in these samples represent the node and not the PS as discussed by O’Rahilly^5^. One interpretation was that in humans, the primitive node forms prior to streak formation although this is unlikely to be the case as in both mouse and chick the node forms after the PS. The presence of a node-like structure recognized in these CS6b specimens most likely indicated that a PS has already formed, although it could not be visually detected. In summary these discussions highlight the difficulty in precisely staging the onset of gastrulation in humans given the limited historical samples available at this stage of development and speak to the need for detailed characterization in multiple other model systems.

## Transcriptional analysis of human gastrulation

Recently we were fortunate to receive a human embryo in the process of gastrulation^45^. Given the clearly visible morphological features, such as a node, extended primitive streak and prechordal plate, we were able to stage this embryo as CS7. In contrast to the historical specimens discussed above which were analysed post-fixation, we were able to assess the dimensions of a fresh, unfixed, specimen. The span of the complete embryo from amnion to definitive yolk sac was 1.66mm. The embryonic disk extended 1.35mm from rostral to caudal edge and was 0.98mm wide, while the primitive streak was 0.67mm in length (Figure 2). On the basis of fixed specimens in the Carnegie collection, O’Rahilly concluded that the embryonic disk at CS7 was generally between 0.3 to 0.7mm in length along the rostral to caudal axis, but could extend to 1mm. The primitive streak was recorded to be between 0.1 – 0.37mm occupying around 50% of the length of the embryonic disk from the caudal edge of the embryonic disk. The dimensions of our specimen were somewhat larger than those of previously described samples, which might be due to the historical samples having undergone shrinkage which can occur during fixation depending on fixative used^46^. Given the range of sizes described historically, further unfixed specimens will be needed to determine if these differences reflect biological variation or are technical.

Over the last decade there has been a rapid increase in the ability to characterize single cells at both the transcriptomic and anatomical level. Large single-cell transcriptomic datasets now exist covering early embryo development at high-temporal resolution in multiple model species including zebrafish^47^, Xenopus^48^, mouse^49^ and non-human primates^50^. This has enabled the characterization of progenitor types based on 1000s of genes as well as the temporal dynamics of gene expression during development. Given the sample we received was fresh, we took the opportunity to perform a single cell transcriptional characterization of the gastrula, which allowed us to define the cell types present and investigate gene expression dynamics during human gastrulation.

## Species Specific Similarities and Differences

During gastrulation in mouse and other model species, epithelial cells of the epiblast undergo an epithelial to mesenchymal transformation (EMT) by downregulating adherens junction molecules such as E-Cadherin (CDH1) so they can delaminate from the epiblast and migrate away as mesenchymal cells^51–53^. Although studies using in vitro models of differentiation have identified similar processes occurring in human cell lines^8^, historically it has been impossible to look at this process in vivo. We used the CS7 human sample to compare cells actively undergoing gastrulation in vivo between species. Given the mouse is the leading model for studying mammalian gastrulation, we examined and compared the transcriptional changes which occur during gastrulation in both the human and the mouse. We observed many similarities in transcriptional changes, such as a decrease in CDH1 during transition from epiblast to nascent mesoderm, transient expression of TBXT and increasing SNAI1 during nascent mesoderm formation^45^. However, there were also some notable differences. One example was in the expression of the zinc-finger transcription factor SNAI2 (Slug), a regulator of EMT. SNAI2 levels increased dramatically during nascent mesoderm formation in the human. However, SNAI2 was not detected during this transition in the mouse. The lack of requirement for SNAI2 during mouse gastrulation is consistent with the viability of SNAI2 null mice^54^. By contrast, in the chick, as in the human, SNAI2 is expressed within the PS and interfering in its expression results in the impaired emergence of mesoderm from the PS^55^. Together this suggests that unlike in the mouse, in human, SNAI2 may play a role in regulating EMT during gastrulation.

As well as differences in core transcription factors, we also detected difference in signaling molecules. In the mouse, the expression of various signaling molecules is crucial for EMT, germ layer specification and migration^56–59^. TDGF1, a NODAL co-receptor essential for normal mesodermal patterning, shows an increase in expression during primitive streak and nascent mesoderm formation in the mouse. In contrast, in the human gastrula, TDGF1 expression showed the opposite trend, decreasing as nascent mesoderm formed. FGF8 is the only known FGF directly required for gastrulation^60^ in the mouse, playing a particularly important role in the migration of cells away from the PS^53^. By contrast, FGF8 was completely absent during the transition from Epiblast to Nascent Mesoderm in human. Other FGF members are expressed during this transition however, including FGF4 (which is also expressed in the mouse), and FGF2, which is not expressed or required for gastrulation in the mouse^61,62^. Interestingly treatment of in vitro cultured mouse epiblasts with FGF2 resulted in the altered fate of these cells from ectoderm to mesoderm^63^, pointing to a degree of redundancy in function between these FGFs.

Together, this comparison indicates that there is broad conservation of several molecular players in human and mouse gastrulation, such as the involvement of the SNAIL/SLUG family of transcriptional repressors and the influence of FGF/MEK-dependent EMT. However, the specific members of these families vary between humans and mice, it will therefore be interesting to examine the underlying reason for these differences.

**Figure 3:**
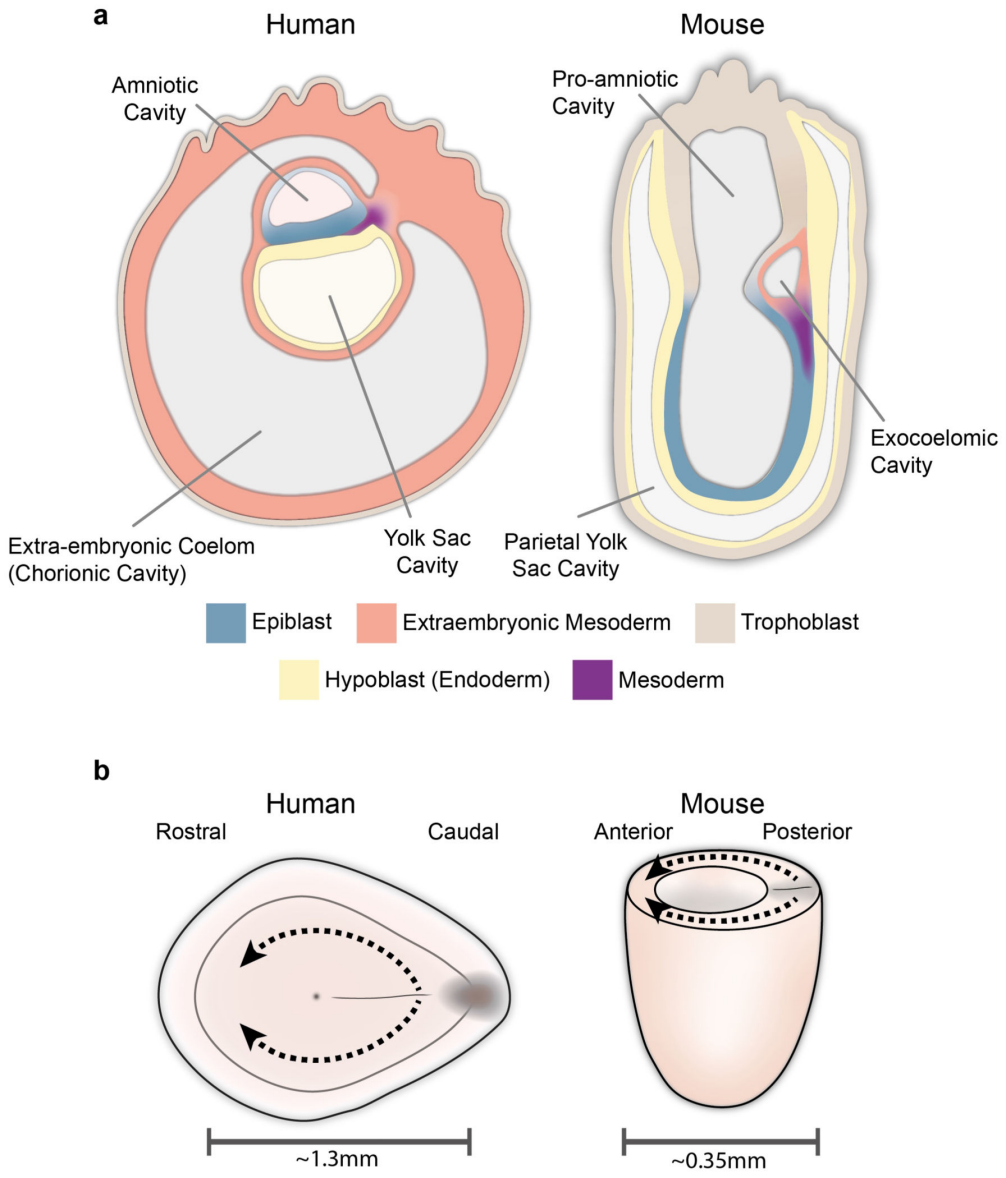
Morphological differences between human and mouse a, Mid-sagittal view of a human (CS6b/7) and mouse (E 7.0) embryo at the onset of gastrulation highlighting the morphological differences as well as corresponding tissue types. In the human, the endoderm adjacent to the epiblast is the Hypoblast but extends to line the extraembryonic secondary yolk sac. In the mouse, the endoderm surrounding the egg cylinder is termed visceral endoderm but extends to line the trophoblast and is termed parietal endoderm. b, Schematic diagrams highlighting differences in the size of human and mouse embryos at comparable stages of development. The dotted lines represent mesoderm migration, which due to both size and morphology is further in the human than in the mouse.

One reason for these differences could relate to species differences in embryo morphology. Both the human and chick gastrula develop as a disk, with mesoderm cells emerging through the streak in the caudal portion of the embryo and migrating in a rostral direction^64^. In contrast, the mouse embryo is cylindrical in shape, with mesoderm cells migrating along the lateral sides of this cylinder^65^ (Figure 3a). Detailed fate mapping experiments in the chick^66–68^ and mouse^69–71^ show that despite their different shapes and the paths that migrating mesodermal cells have to take to reach their destination, the fate of cells emerging from different rostro-caudal positions along the streak is broadly similar in both species. Such difference in the shape of the embryo might be the reason there are also differences in the expression of molecules that may have a role in coordinating cell migration. On could speculate that, particularly in the case of secreted signaling molecules, the different family members expressed might have, for example, different diffusion properties tuned to the specific morphology of the embryo, resulting in equivalent outcomes despite the differences in overall morphology.

Furthermore, differences in the geometry and size of the embryo could influence the signaling gradients experienced by cells in the embryo. For example, the mouse embryo at early pre-headfold stage (EPHF)^72^ is cylindrical and, at the embryonic-extraembryonic boundary, approximately 330 to 400 microns wide^72,73^ between the rostral and caudal extremes. In humans at an equivalent stage, the embryo is a disk and extends approximately 1350 microns from the rostral to caudal edge of the embryo^45^, highlighting the increased distance across which diffusible signals might have to act (Figure 3b). Furthermore, given the cylindrical shape of the mouse embryo at these stages, the part of the streak closest to the rostral cardiac forming region is the proximal (caudal) end of the streak. In contrast, in the human embryonic disk, it is the rostral end of the streak that is closest to the cardiac crescent. Differences in morphology could also affect the forces experienced as cells emerge through the primitive streak. In the chick, which also forms as a disk like the human, a tensile ring forms at the margin between the embryonic and extraembryonic regions^74^, which generates forces that can drive the vortex-like “polonaise” movements that accompany primitive streak formation^75^. Given the cylindrical nature of the mouse embryo, it is possible that the distribution of mechanical forces generated during primitive streak formation is different, resulting in delaminating cells in the mouse streak being subject to a different mechanical, as well chemical signaling milieu.

## Developmental trajectories during human gastrulation

Cell lineage reflects the developmental history of a particular tissue, characterizing the cellular ancestry from progenitor to mature cell type. In model systems, this developmental history has been, to different extents, reasonably well characterised. For example, in *C. elegans*, the lineage of all cells has been mapped facilitated by the relatively small number of cells (959 in the adult hermaphrodite), determinate development, and transparent body enabling cell dynamics to be observed^76^. However, the lineal relationship between key cell types in the human embryo remains unclear. Examining cellular ancestry in the human is important given the species-specific differences in the formation of some key cell types. One example of this is the extraembryonic mesoderm which is of interest given it is the source of primitive blood in the developing embryo. In the mouse, extraembryonic mesoderm is observed only after the onset of gastrulation, and is derived from epiblast cells that have delaminated through the caudal primitive streak at early to mid-streak stages^69,70,77^. In humans, and non-human primate embryos, extraembryonic mesoderm can already be detected at around Carnegie Stage 5 (~Day 11), prior to primitive streak formation^5^. This tissue has been described as forming a “fine loose mesh work” which fills the space between the exocoelomic membrane and the inner aspect of the trophoblast^4^. The appearance of extraembryonic mesoderm prior to the onset of gastrulation suggests a non-streak and potentially, a non-epiblast contribution to the extraembryonic mesoderm in humans. While there is evidence to indicate that in primates, the extra-embryonic mesoderm might have a contribution from the hypoblast of the bilaminar disk stage embryo^78^, the precise origin of the cells seen prior to the primitive streak remains unclear^79^, as does the relative contribution to the extraembryonic mesoderm of cells emerging through the streak during gastrulation.

Our recent single cell transcriptomic analysis of a CS 7 embryo showed that extra-embryonic mesoderm (collected predominantly from the yolk-sac) was transcriptionally distinct from embryonic mesoderm. Diffusion mapping, which orders cells based on their transcriptional state, showed a continuous trajectory from epiblast to the most advanced embryonic derived mesoderm, the ‘advanced mesoderm’. This trajectory likely reflected the extent of their differentiation and the ‘age’ of cells, based on how far in the past of this sample they had emerged from the epiblast. Interestingly, this trajectory was not continuous with the Extraembryonic mesoderm population, suggesting that the extraembryonic mesoderm may not be as closely related as other mesoderm populations.

Such differences between the mouse and human highlights the need for further insight into the clonal relationship between cell types in the human. Traditionally lineage analysis in model species has relied on genetic lineage labels to tag individual cells and their descendants. Such genetic labels have varied from LacZ^80^ and fluorescent reporters^81^ to more recent approaches such as CRISPR-based genetic scars and DNA barcodes^82–84^. However these approaches require genetic engineering and cannot be applied to *in vivo* human tissue. Therefore approaches have been developed which use somatic mutations^85,86^ or variations in mitochondrial DNA^87^ to reconstruct cell lineage. The use of somatic mutations to reconstruct lineal relationships has recently provided data supporting a hypoblast-derived origin for extraembryonic mesoderm^88^. Although this study analyses a single Carnegie Stage 23 (8 weeks post conception) embryo, it highlights the exciting applications of sequencing technologies to important questions in human developmental biology. As these technologies become more widespread and ethically approved human samples become available, our insight into human development will rapidly increase.

## The importance and limitations of ‘marker’ genes

To accurately annotate transcriptional datasets and understand developmental dynamics, we rely on prior knowledge of the cell type specific expression of marker genes. This is a particular challenge in the human when samples are limited, increasing our reliance on expression profiles from model systems such as the mouse. This challenge is even more acute when studying gastrulation, given the dynamic nature of the process and rapid changes in gene expression during differentiation and maturation. While we could detect some interspecies differences in expression, overall we found that molecular markers were conserved between human and species such as the mouse and non-human primates^45^.

When using markers to annotate cell types in transcriptomic studies, it is important to not conflate cell state, with cell fate. Current transcriptomic technology captures a freeze-frame of the transcriptional state of cells at the time the sample was collected. This snapshot is obviously a limited view of a dynamic, changing transcriptional landscape along which progenitor cells travel as they are specified and then commit to a particular fate. While progenitors fated to differentiate into a particular cell type may start to express markers of that cell type, this is generally not sufficient to commit them irreversibly to that fate. This is particularly the case in the early gastrula, given the extensive cell migratory activity and evidence from heterotopic transplantation experiments of the plasticity of mesodermal progenitors^89^. In the case of transcriptomic studies in model organisms such as the mouse, one can generally use experimental approaches to lineage label cells belonging to specific transcriptional clusters to determine their fate^90^, which is not an option in studies of early human embryos.

The algorithms used for the unbiased hierarchical clustering of cells based on their transcriptome generally rely on differences in the expression of hundreds of highly variable genes in the data set. When annotating clusters, we per force can only consider the subset of these genes that have been characterized as ‘markers’ in the literature, which can lead to marker genes taking on a life of their own, to the detriment of accurate annotation. The variable expression of specific marker genes within transcriptional clusters can also make cell type identification challenging and can lead to over-simplified conclusions. This was highlighted in the mesodermal cells of the CS 7 gastrula, which expressed a mixture of ‘markers’ of specific mesodermal sub-types. By exploring the co-expression of marker signatures within individual cells we could determine that markers of mesodermal sub-types (e.g. lateral plate mesoderm, paraxial mesoderm etc.) were either seen only in a subset of a cluster or spanned multiple clusters. For example, co-expression of TBX6 and MSGN1, which marks pre-somitic mesoderm^91^, was only detected in a subset of the nascent mesoderm cluster. In contrast, co-expression of HAND1 and GATA6, which marks lateral-plate mesoderm, could be detected in multiple mesoderm clusters including the nascent mesoderm cluster, within which cells with a paraxial mesoderm signature could also be identified. This led us to conclude that the different mesodermal sub types had not clearly emerged yet at this stage of gastrulation, and that mesodermal cells were transcriptionally clustering on maturation status.

Another challenge when using marker genes is defining cell types at boundaries, that may represent transitional populations that have aspects of the transcriptional signatures of two or more cell types. Examples of such boundaries include those between the yolk sac endoderm and hypoblast, lateral plate mesoderm, somatic and extraembryonic mesoderm as well as the amniotic ectoderm and surface ectoderm. This challenge was highlighted when we tried to annotate the ectoderm cell types in the CS7 human gastrula, that included both embryonic as well as extraembryonic ectoderm (of the amnion). There is extensive overlap in the expression of ectodermal markers such as DLX5, TFAP2C and GATA3, between these two cell types^92,93^. Further sub clustering of the ectoderm population, revealed two transcriptionally distinct cell types, one that could be annotated as amniotic ectoderm based on expression of VTCN1^94^ and another population that we annotated as non-neural ectoderm. However as the non-neural ectoderm forms at a boundary between the epiblast and amniotic ectoderm we could not determine whether this population represented embryonic cells such as surface ectoderm, or immature cells in the process of differentiation into amniotic ectoderm.

Similar ambiguity exists in categorizing the extra-embryonic mesoderm of the amnion and yolk sac, that are continuous with each other (Figure 3a). In early head fold gastrulating mouse embryos (approximately E7.5 to E8.0) *Periostin* is commonly used as a marker of amniotic mesoderm ^95,96^. However, its expression extends beyond the amniotic mesoderm into the mesoderm overlying the yolk sac (Figure 3 in ^95^), making it a marker more generally of extra-embryonic mesoderm. We are able to infer that the *Periostin* expressing extra-embryonic mesoderm transcriptional cluster in the CS 7 embryo likely represented yolk sac mesoderm because the majority of cells in this transcriptional cluster were collected from the yolk sac. This demonstrates the advantage of being able to leverage even simple anatomical information in annotating cell types. This required us to sub-dissect the CS 7 gastrula into three broad anatomical domains (yolk sac, rostral and caudal embryonic disc) prior to disaggregating to single cells. Such anatomical information was also important in distinguishing the yolk sac endoderm from the contiguous hypoblast cells.

If one does not start out knowing the anatomical origin of sequenced cells, one can also go in the other direction, and visualize the anatomical distribution of cells belonging to transcriptional clusters using the intersectional expression of multiple markers of that cluster. The development of methodologies, such as HCR RNA fluorescence in situ hybridization^97,98^ now allows multiplexed detection of the expression of several genes at single-cell resolution in whole mount samples. While only a handful of genes can be assessed, the 3D single-cell resolution data generated allows spatial expression profiles to be precisely characterised. This high-resolution characterisation of expression combined with transcriptomics allows one to very accurately map transcriptional clusters, providing important biological insight^90^. At the other end of the spectrum, spatial transcriptomics approaches are rapidly expanding, providing methodologies that enable the spatial profiling of 1000’s of genes, but typically on sectioned tissue, making reconstruction of full 3D information difficult. Nevertheless, they provide valuable information about the anatomical location of sequenced cells. Application of such single cell transcriptomic technologies to early human embryos will allow us to gain a better understanding of how cell type specific molecular profiles vary depending on spatial location during gastrulation and development.

## Conclusion

Our understanding of human gastrulation as it occurs *in utero* is limited, in large part due to the extreme rarity of obtaining such samples for study. For example, our study of the CS7 embryo was based on a single sample. We showed by several measures that it was very likely ‘normal’ (morphologically comparable to other fixed samples, euploid, distribution of cell-cycle phases and of normal genomic integrity), but the singular nature of the sample imposes obvious limits on the extent to which we can generalize, and also, does not capture any natural variation among human embryos. The recent development of various *in vitro* models of gastrulation^9–11,13–16^ therefore present exciting opportunities for studying this process. It is important that these models accurately recapitulate development occurring *in utereo* and our molecular characterisation of cell types in the gastrula provides a means to begin to benchmark these different model systems^99,100^.

Research into human gastrulation now appears poised to enter a golden age, thanks not only to methodological breakthroughs in our ability to culture human embryos but also due to technical advances in single cell sequencing and high-resolution time-lapse imaging^101^, that allow analyses to be conducted at previously impossible levels of detail. The final hurdle in ushering in this golden age lies in the difficult ethical and scientific debate surrounding the relevance of the so called ‘14-day rule’. Recently the International Society for Stem Cell Research (ISSCR) updated its guidelines regarding the culture of human embryos^102^, potentially paving the way in the near future for experiments on human embryos cultured to gastrulation stages, allowing us to gain an even better understanding of this critical but still mysterious process, that lays the foundation for the body plan of the fetus.
